# β-Lapachone Induces Acute Oxidative Stress in Rat Primary Astrocyte Cultures that is Terminated by the NQO1-Inhibitor Dicoumarol

**DOI:** 10.1007/s11064-020-03104-0

**Published:** 2020-08-13

**Authors:** Johann Steinmeier, Sophie Kube, Gabriele Karger, Eric Ehrke, Ralf Dringen

**Affiliations:** 1 Centre for Biomolecular Interactions Bremen, Faculty 2 (Biology/Chemistry), University of Bremen, P.O. Box 330440, 28334 Bremen, Germany; 2 Centre for Environmental Research and Sustainable Technology, University of Bremen, Bremen, Germany

**Keywords:** Astrocytes, Dicoumarol, GSSG, NQO1, Oxidative stress

## Abstract

β-lapachone (β-lap) is reduced in tumor cells by the enzyme NAD(P)H: quinone acceptor oxidoreductase 1 (NQO1) to a labile hydroquinone which spontaneously reoxidises to β-lap, thereby generating reactive oxygen species (ROS) and oxidative stress. To test for the consequences of an acute exposure of brain cells to β-lap, cultured primary rat astrocytes were incubated with β-lap for up to 4 h. The presence of β-lap in concentrations of up to 10 µM had no detectable adverse consequences, while higher concentrations of β-lap compromised the cell viability and the metabolism of astrocytes in a concentration- and time-dependent manner with half-maximal effects observed for around 15 µM β-lap after a 4 h incubation. Exposure of astrocytes to β-lap caused already within 5 min a severe increase in the cellular production of ROS as well as a rapid oxidation of glutathione (GSH) to glutathione disulfide (GSSG). The transient cellular accumulation of GSSG was followed by GSSG export. The β-lap-induced ROS production and GSSG accumulation were completely prevented in the presence of the NQO1 inhibitor dicoumarol. In addition, application of dicoumarol to β-lap-exposed astrocytes caused rapid regeneration of the normal high cellular GSH to GSSG ratio. These results demonstrate that application of β-lap to cultured astrocytes causes acute oxidative stress that depends on the activity of NQO1. The sequential application of β-lap and dicoumarol to rapidly induce and terminate oxidative stress, respectively, is a suitable experimental paradigm to study consequences of a defined period of acute oxidative stress in NQO1-expressing cells.

## Introduction

**Fig. 1:**
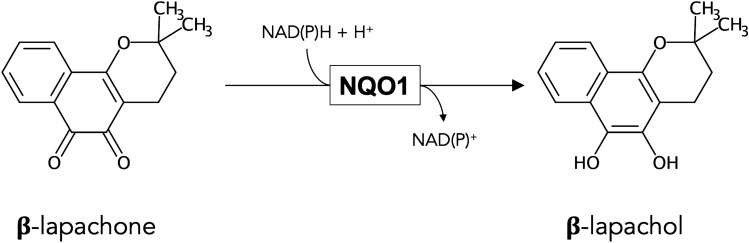
β-lapachone and β-lapachol. β-lapachone (quinone-form) can be reduced in a two-electron reaction to β-lapachol (hydroquinone-form) by NAD(P)H: quinone acceptor oxidoreductase 1 (NQO1)

The quinone beta-lapachone (β-lap, clinical names: ARQ761 or ARQ501) has been extracted from the bark of the lapacho tree and is known to have various beneficial effects [1, 2]. For example, it has frequently been applied in anti-cancer studies on cells and tissues, targeting for example prostate cancer [3], pancreatic cancer [4], lung cancer [5], breast cancer [6], melanoma [7] or astrocyte-like glioma [8]. The proposed mechanism of the antitumor action is the activation of β-lap by NAD(P)H: quinone acceptor oxidoreductase 1 (NQO1, EC 1.6.99.2) (Fig. 1), which catalyses the obligatory two-electron reduction of quinones [9, 10]. NQO1-mediated catalysis is widely regarded as beneficial, as it avoids an undesirable one-electron reduction that is directly associated with radical formation and oxidative stress [9, 11, 12] and alleviates clearance by phase II enzymes [9]. However, the hydroquinone form of β-lap (β-lapachol, Fig. 1) that is generated by NQO1-mediated reduction is labile and auto-oxidizes quickly in two distinct one-electron steps [1, 10], thereby starting a futile cycle that regenerates the quinone β-lap by producing intracellular ROS which leads to oxidative stress and cell toxicity [2, 13]. This NQO1-dependent cycling of β-lap appears to be especially prominent in cancer cells, since such cells are reported to contain higher activities of NQO1 than non-cancerous cells, which supports the potential use of β-lap as an anti-cancer drug [1, 14].

In brain, astrocytes are the first parenchymal cells behind the blood–brain barrier [15, 16] and are therefore considered as the first line of defence against xenobiotics causing oxidative stress [17]. Astrocytes possess an elaborated antioxidative defence system, that includes high activities of antioxidative enzymes and millimolar concentrations of the isopeptide glutathione (GSH) [18–20]. Glutathione is a crucial antioxidant [11, 17, 21] that plays a key role in the maintenance of the cellular thiol-redox potential and is substrate of enzymes involved in the defence of cells against oxidative stress and xenobiotics [17]. The product of the oxidation of GSH by glutathione peroxidases (GPx, EC 1.11.1.9) is glutathione disulfide (GSSG), which is quickly recycled in viable cells to GSH by the NADPH-consuming glutathione reductase (GR, EC 1.8.1.7) [17, 22]. In addition, GSH can be conjugated to xenobiotics by glutathione-*S*-transferases to GSH-conjugates [17]. In unstressed astrocytes, hardly any GSSG is detectable, but several compounds have been described to induce GSH oxidation to GSSG which is subsequently exported from the cells, including peroxides [23], catecholamines [24] as well as quinones such as menadione [25, 26]. Depletion of cellular GSH in astrocytes was also reported for cells that had been exposed to alkylating substances like iodoacetate [27], 3-bromopyruvate [28] or dialkyl-fumarates [29]. In cultured astrocytes, the export of GSH, GSSG and GSH-conjugates is mediated by the ATP-dependent multidrug resistance protein 1 (Mrp1) [30–34].

So far little information is available on the consequences of an exposure of brain cells to β-lap. Chronic exposure to 1 µM β-lap has been reported to induce in primary rat astrocytes the expression of protective and antioxidative enzymes (e.g. NQO1, catalase), to increase cellular GSH contents and to protect the cells against hydrogen peroxide-induced oxidative stress [35]. These results are consistent with a recent report on the neuroprotective potential of β-lap in a MPTP-induced Parkinson’s disease mouse model which involves the upregulation of Nrf2-controlled pathways in astrocytes [36]. Even lower concentrations of β-lap than 1 µM have been reported to activate glutamate dehydrogenase and to attenuate iodoacetate-induced toxicity in cultures of cortical neurons or astrocytes [37]. Moreover, exposure of rat primary microglia to β-lap lowered nitrite and ROS levels and increased the expression of NQO1 [38].

Astrocytes have been reported to contain substantial activity of NQO1 [35, 39] and should therefore encounter NQO1-mediated ROS production and oxidative stress after application of β-lap. Here we report that acute exposure of cultured astrocytes to low micromolar concentrations of β-lap caused rapid cellular ROS formation and GSH oxidation which is followed by GSSG export and a delayed impairment in cell metabolism and cell viability. All these adverse effects of a β-lap application to astrocytes were completely prevented by the NQO1 inhibitor dicoumarol, demonstrating that the presence of this enzyme makes cultured astrocytes sensitive towards β-lap. In addition, the dependence of β-lap-induced oxidative stress on the activity of NQO1 makes the sequential application of β-lap and dicoumarol a suitable experimental system to rapidly induce (application of β-lap) and terminate (addition of dicoumarol) an acute oxidative stress condition in cultured NQO1-expressing cells.

## Material and Methods

### Material

β-lap (ab141097) was purchased from Abcam (Berlin, Germany) and dicoumarol (M1390) from Sigma-Aldrich (Steinheim, Germany). Dulbecco’s modified Eagle’s medium (DMEM with 25 mM glucose) and penicillin/streptomycin solution was obtained from Gibco (Darmstadt, Germany) and fetal calf serum from Biochrom (Berlin, Germany). Bovine serum albumin (BSA), NADPH, NADH and sulfosalicylic acid were from AppliChem (Darmstadt, Germany) and the enzyme glutathione reductase was purchased from Roche Diagnostics (Mannheim, Germany) and from Sigma-Aldrich (Steinheim, Germany). All other chemicals of the highest purity available were purchased from Merck (Darmstadt, Germany), Sigma-Aldrich (Steinheim, Germany), Fluka (Buchs, Switzerland), Dojindo (Munich, Germany), Roche (Mannheim, Germany), Roth (Karlsruhe, Germany) or Riedel-de Haën (Seelze, Germany). Sterile cell culture plates and non-sterile normal and black 96-well microtiter plates were from Sarstedt (Nümbrecht, Germany).

### Astrocyte Cultures

Primary astrocyte cultures were prepared from whole brains of new-born Wistar rats after decapitation as previously described [40]. Wistar rats were purchased from Envigo RMS (Rossdorf, Germany). Animals were treated in accordance with the animal regulations of the University of Bremen and of the state of Bremen (Germany). For the preparation of primary cultures a special institutional ethical approval was not required. Primary astrocyte cultures contain mainly GFAP-expressing astrocytes and only minor amounts of oligodendrocytes and microglial cells [40, 41]. Harvested cells were seeded in a density of 300,000 viable cells in 1 mL of culture medium (90% DMEM, 10% FCS, 1 mM pyruvate, 18 U/mL penicillin G and 18 µg/mL streptomycin sulfate) in wells of a 24-well plate and incubated for at least two weeks at 37 °C with 10% CO_2_ in the humidified atmosphere of a cell incubator (Sanyo, Japan). Experiments were performed on confluent cultures of an age between 14 and 28 days. The only exception from this was one culture used for one replication of the experiment shown in Fig. 8, which was 39 days old. Every 7 days and one day before an experiment the culture medium of the cells was renewed.

### Cell Incubations

Astrocyte cultures were washed with 0.5 mL pre-warmed (37 °C) incubation buffer (IB; 20 mM HEPES, 5 mM D-glucose, 145 mM NaCl, 5.4 mM KCl, 1.8 mM CaCl_2_, 1 mM MgCl_2_, 0.8 mM Na_2_HPO_4_, pH 7.4) and incubated at 37 °C with 200 µL IB in the absence or the presence of the other compounds indicated in the figure legends in the humidified atmosphere (without CO_2_) of a cell incubator (Sanyo, Japan). After the given incubation periods, media were harvested and the cells were washed with 1 mL ice-cold (4 °C) phosphate-buffered saline (PBS; 10 mM potassium phosphate buffer, pH 7.4, containing 150 mM NaCl) for subsequent analysis of cellular and extracellular components.

### Tests for Cell Viability

The viability of astrocyte cultures after a given treatment was determined by testing for membrane integrity by measuring the extracellular lactate dehydrogenase (LDH, EC 1.1.1.27) activity and by nuclear propidium iodide staining as described previously [40]. Extracellular LDH was determined in 10 µL media samples. The activity is given as the percentage of the initial cellular LDH activity of control cells that had been lysed in 200 µL IB containing 1% (v/v) Triton X-100. For propidium iodide staining the treated cells were washed twice with 1 mL prewarmed (37 °C) IB, incubated for 15 min at 37 °C with 500 µL propidium iodide staining solution (5 µM propidium iodide plus 10 µM Hoechst 33342 (H33342) in IB) and subsequently washed twice with 1 mL IB before fluorescence images were recorded using a Nikon Eclipse TE2000U fluorescence microscope equipped with a DSQiMc camera and the imaging software NIS-Elements (Nikon, Düsseldorf, Germany). Appropriate filter settings were used for the visualization of the staining with propidium iodide (excitation: 510–560 nm; emission: 590 nm; dichromatic mirror: 575 nm) and H33342 (excitation: 330–380 nm; emission: 435–485 nm; dichromatic mirror: 400 nm). For all images identical microscopic settings and image processing was applied.

### Lactate and Protein Quantification

The extracellular accumulation of lactate during the incubation was measured for 10 µL medium samples by a coupled enzymatic assay system containing LDH and glutamate pyruvate transaminase as described previously in detail [40, 42]. The initial protein content of the astrocyte cultures investigated was determined according to the Lowry method [43] using bovine serum albumin as standard protein.

### Quantification of Glutathione Contents

Total glutathione (GSx = amount of GSH plus twice the amount of GSSG) and GSSG were quantified for cell lysates and medium samples by a colorimetric enzymatic cycling assay as previously described in detail [40] which is based on the method originally described by Tietze [44]. The washed cells were lysed with 200 µL 1% (w/v) sulfosalicylic acid at 4 °C and the lysates were used to determine the contents of GSx and GSSG. Quantification of the extracellular contents of GSx and GSSG was performed for 20 µL of a 1:1 mixture of the harvested medium and 1% (w/v) sulfosalicylic acid.

### Determination of ROS Production

For quantification of cellular ROS production, the non-fluorescent dihydrodichlorofluorescein-diacetate (DCFH_2_-DA) was applied which is trapped within cells by enzymatic deacetylation to the DCFH_2_ which can be oxidized in cells by ROS to the fluorescent dichlorofluorescein (DCF) [45]. Cultured astrocytes were washed with 1 mL of IB and loaded with the non-fluorescent dye by a 30 min incubation with 200 µL 50 µM DCFH_2_-DA in IB at 37 °C. Subsequently, the cells were washed twice with 1 mL of IB and incubated for 5 min at 37 °C with 200 µL IB containing β-lap in the indicated concentrations in the absence or the presence of the NQO1 inhibitor dicoumarol. Finally, the cells were lysed in 400 µL ice-cold (4 °C) hypotonic potassium phosphate buffer (20 mM, pH 7.4) on ice for 10 min in the dark and the harvested lysates were centrifuged for 1 min at 12,300 g. DCF fluorescence (excitation at 485 nm, emission at 520 nm) was determined for 200 µL of lysate supernatant in wells of a black microtiter plate in a plate reader (Fluoroskan Ascent FL, Thermo Fisher Scientific, Schwerte, Germany).

### Determination of Extracellular WST1 Formazan Production

The water-soluble tetrazolium salt 1 (WST1) is a membrane impermeable substance that can be reduced in cell cultures by membrane permeable electron cyclers to form a water-soluble formazan product [46]. To test whether β-lap can serve as electron cycler for WST1 reduction, cultured astrocytes were washed once with 1 mL of IB and then incubated in 200 µL of IB containing 5 mM glucose, 20 µM β-lap and 400 µM WST1 in the absence or the presence of other compounds which are listed in the legend of Fig. 8. After 10 min or 30 min of incubation, 50 µL of incubation solution were harvested, diluted with 150 µL of water in wells of a microtiter plate and the absorbance of the WST1 formazan generated was measured at 450 nm in a plate reader. The concentration of WST1 formazan in the incubation medium was calculated from the absorbance by using the extinction coefficient of 35.2 mM^−1^ × cm^−1^ [46].

### Presentation of Data and Statistical Analysis

The quantitative data presented are means ± standard deviations (SD) of values that were obtained in experiments performed on three independently prepared astrocyte cultures. For analysis of the significance of differences between three or more sets of data analysis of variance (ANOVA) followed by a Bonferroni post-hoc test was used, and statistically significant differences are indicated by asterisks which are written in the colours of the respective symbols. p values above 0.05 were considered as not significant. Statistical analysis was performed using the software GraphPad InStat version 3.10. The images shown for propidium iodide staining of cultured astrocytes are derived form a representative experiment that was reproduced twice on independently prepared astrocyte cultures with similar outcome.

## Results

### β-Lap Impairs Cell Metabolism and Cell Viability of Cultured Astrocytes

**Fig. 2:**
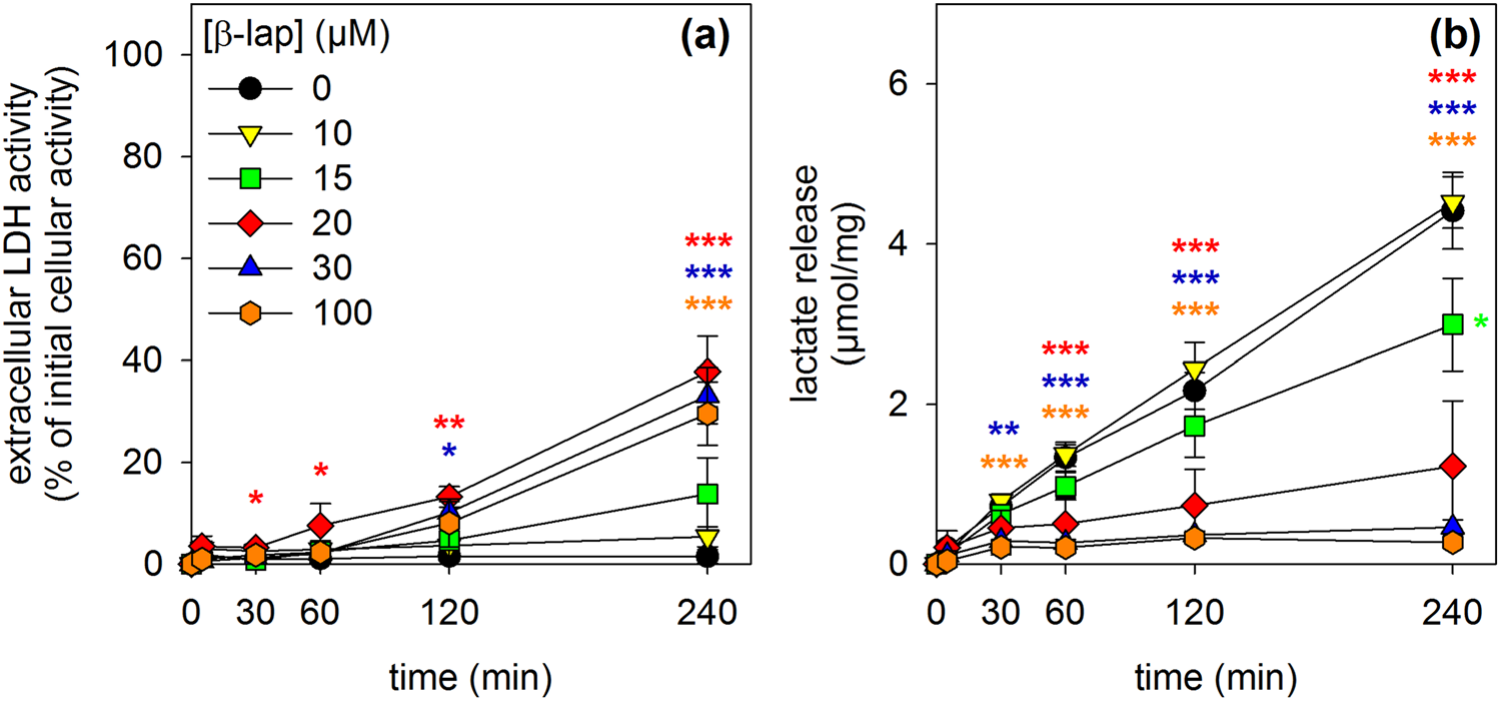
Time- and concentration-dependent effects of β-lap on the viability and the lactate release of primary astrocytes. The cells were incubated with the indicated concentrations of β-lap for up to 240 min. For the indicated time points, the extracellular LDH activity (**a**) and the extracellular lactate content (**b**) were determined. The protein content of the cultures was 137 ± 5 µg/well. The data shown are means ± SD of values obtained in 3 experiments performed on independently prepared cultures (n = 3). Significant differences (ANOVA) of data compared to the data obtained for control cells (incubation without β-lap) are indicated by asterisks written in the colours of the respective symbols (*p < 0.05, **p < 0.01, ***p < 0.001)

**Fig. 3:**
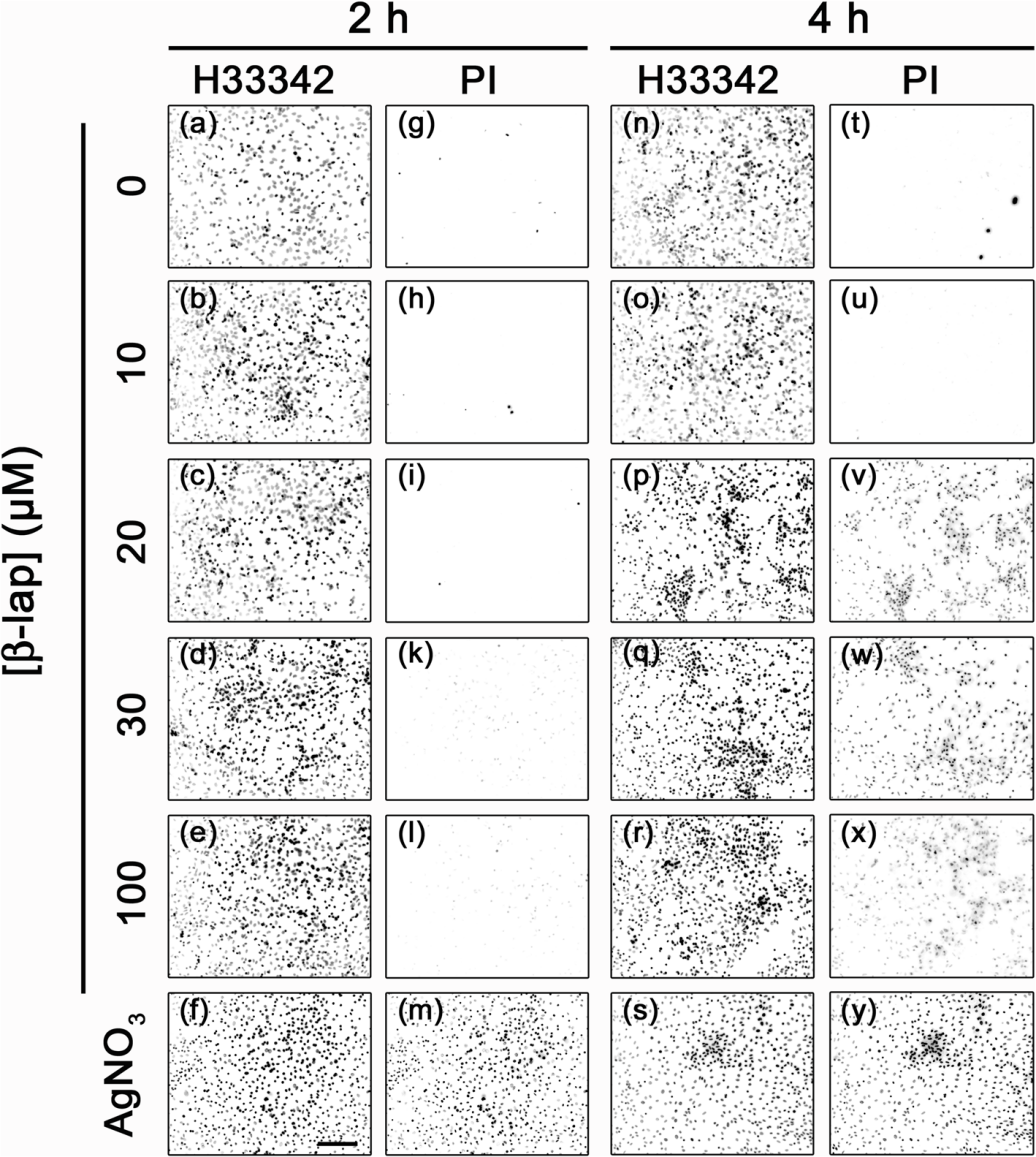
Impairment by β-lap of cell membrane integrity of primary astrocytes. The cells were incubated for 2 or 4 h in IB containing the indicated concentrations of β-lap. As positive control for the loss of cell membrane integrity [60], cells were incubated with 200 µM (2 h) or 100 µM (4 h) AgNO_3_. Shown are cell images of astrocytes that had been stained with H33342 and PI after the given β-lap treatment. The scale bar in panel (**f**) corresponds to 100 µm and applies for all panels. The data shown are from one representative experiment that was repeated twice on independently prepared cultures with similar results

In order to test whether β-lap has adverse consequences on astrocytes, cultured rat astrocytes were exposed to this compound in concentrations of up to 100 µM for up to 4 h and the membrane integrity as well as the lactate accumulation in the incubation medium were determined. Incubations without or with 10 µM β-lap did not cause any increase in the extracellular LDH activity (Fig. 2a), any alteration in the lactate released from the cultures (Fig. 2b) and no increase in the number of PI-positive cells (Fig. 3g, h, t, u) during incubations for up to 4 h. In contrast, β-lap in concentrations above 10 µM impaired the membrane integrity of astrocytes in a time- and concentration-dependent manner as demonstrated by the gradual increase in the extracellular LDH activity (Fig. 2a) and in the extent of PI staining (Fig. 3). Also the glucose metabolism of astrocytes was affected by β-lap as demonstrated by a concentration-dependent decrease in the accumulation of extracellular lactate which was found significant after 4 h of incubation with 15 µM β-lap, while extracellular lactate accumulation was hardly detectable already after a 30 min exposure to 30 µM or 100 µM β-lap (Fig. 2b).

The further analyses of acute effects of β-lap on cultured astrocytes was restricted to incubation periods of up to 120 min as the membrane integrity of the treated cells was not compromised for these incubation periods (Fig. 3).

### β-Lap Induces GSH Oxidation and GSSG Export

**Fig. 4:**
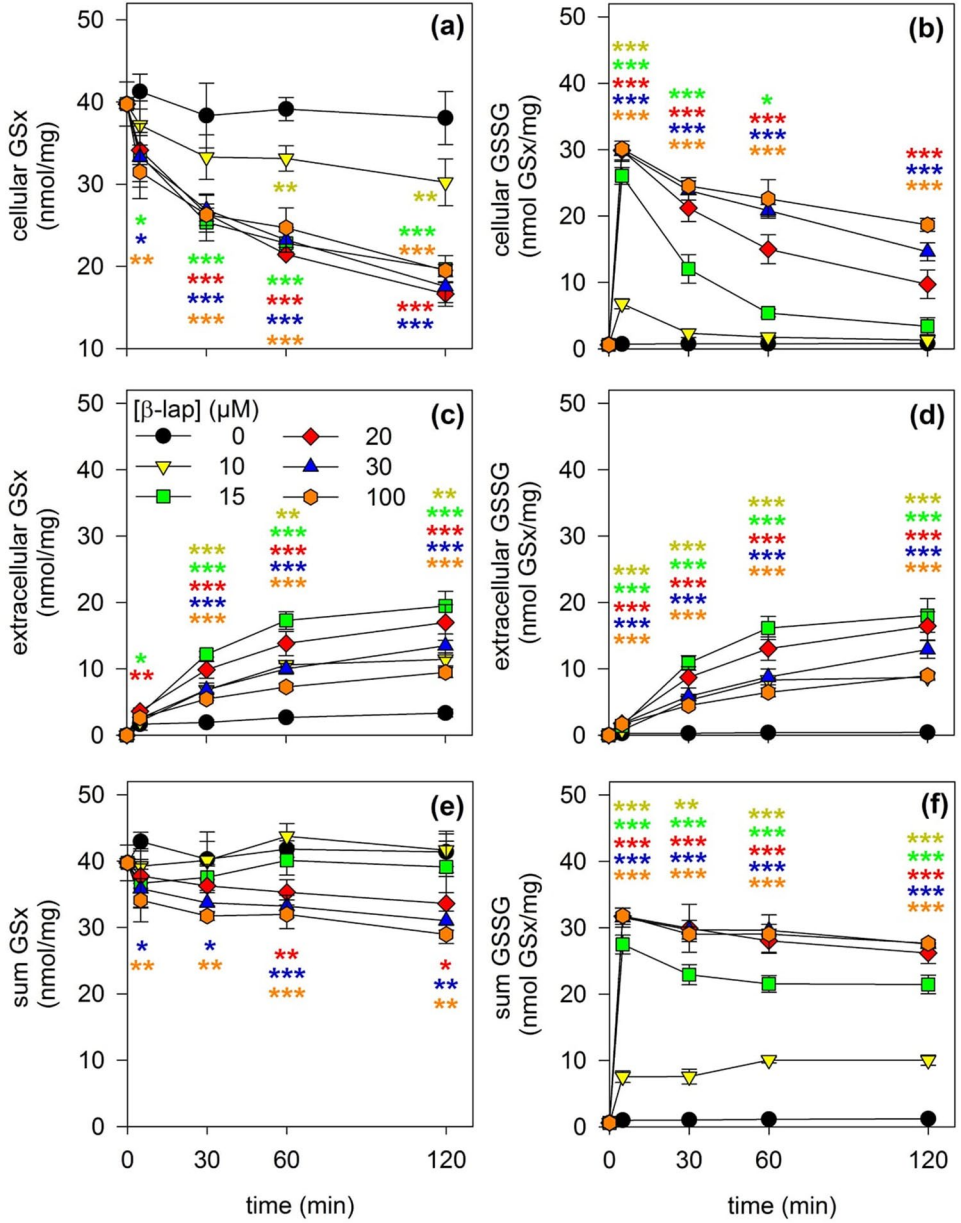
Time- and concentration-dependent effects of a β-lap treatment on the GSx and GSSG contents of astrocyte cultures. The cells were incubated with β-lap in the indicated concentrations for up to 120 min. For the indicated time points the intracellular GSx (**a**) and GSSG (**b**) contents, the extracellular GSx (**c**) and GSSG (**d**) contents as well as the sum of intra- plus extracellular GSx (**e**) and GSSG (**f**) contents of the cultures were determined. The initial specific GSx and GSSG contents of the cultures were 40 ± 3 nmol/mg and 1 ± 0 nmol/mg, respectively. The protein content of the cultures was 137 ± 5 µg/well. The data shown are means ± SD of values obtained in 3 experiments on independently prepared cultures (n = 3). Significant differences as analysed by ANOVA between the values obtained for a given incubation with β-lap compared with the data for control cells (incubation without β-lap) are indicated by asterisks written in the colours of the respective symbols (*p < 0.05, **p < 0.01, ***p < 0.001)

β-lap has been reported to cause oxidative stress in cells [47]. In order to test whether an application of β-lap affects the astrocytic GSH metabolism and the ratio of GSH to GSSG, cultured astrocytes were exposed to β-lap in concentrations of up to 100 µM and the cellular and extracellular contents of GSx and GSSG were determined. During incubation of astrocytes for up to 2 h in the absence of β-lap the cellular GSx content remained almost identical to the initial cellular GSx content (Fig. 4a), a slow increase in the extracellular GSx content was observed (Fig. 4c), the sum of cellular plus extracellular GSx remained constant (Fig. 4e) and only minute amounts of GSSG were found in cells and media (Fig. 4b, d, f). In contrast, already the presence of 10 µM β-lap caused, compared to the control incubation, a time-dependent decrease in the cellular GSx content (Fig. 4a) which was accompanied by an increased extracellular accumulation of GSx (Fig. 4c), as well as a transient appearance of GSSG in the cells within the first 5 min of incubation (Fig. 4b) which was followed by an increased extracellular accumulation of GSSG during the initial 60 min of the incubation (Fig. 4d). Higher concentrations of β-lap than 10 µM further accelerated the loss in cellular GSx, (Fig. 4a), the accumulation of extracellular GSx (Fig. 4c), the appearance of GSSG in cells (Fig. 4b) and the export of GSSG from the cells (Fig. 4d). In addition, with increasing concentration of β-lap, the decline of the initial high GSSG to GSx ratio observed after 5 min of incubation (Fig. 4b) as well as the extracellular GSSG accumulation (Fig. 4d) became slower and a loss in the sum of cellular plus extracellular GSx (Fig. 4e) as well as a strong increase in the sum of cellular plus extracellular GSSG (Fig. 4f) were observed. Almost maximal effects on the GSx and GSSG contents of cultured astrocytes were found for cells that had been exposed to β-lap in a concentration of 20 µM (Fig. 4).

### Dicoumarol Prevents β-Lap-Induced GSSG Formation

The enzyme NQO1 has been proposed to catalyse a two-electron reduction of β-lap [10] which generates the labile reduction product β-lapachol and subsequently ROS and oxidative stress. In order to test for an involvement of NQO1 in the observed β-lap induced changes in astrocytic viability and GSH metabolism, the NQO1-inhibitor dicoumarol [48, 49] was applied in concentrations of 1 or 30 µM.

**Fig. 5:**
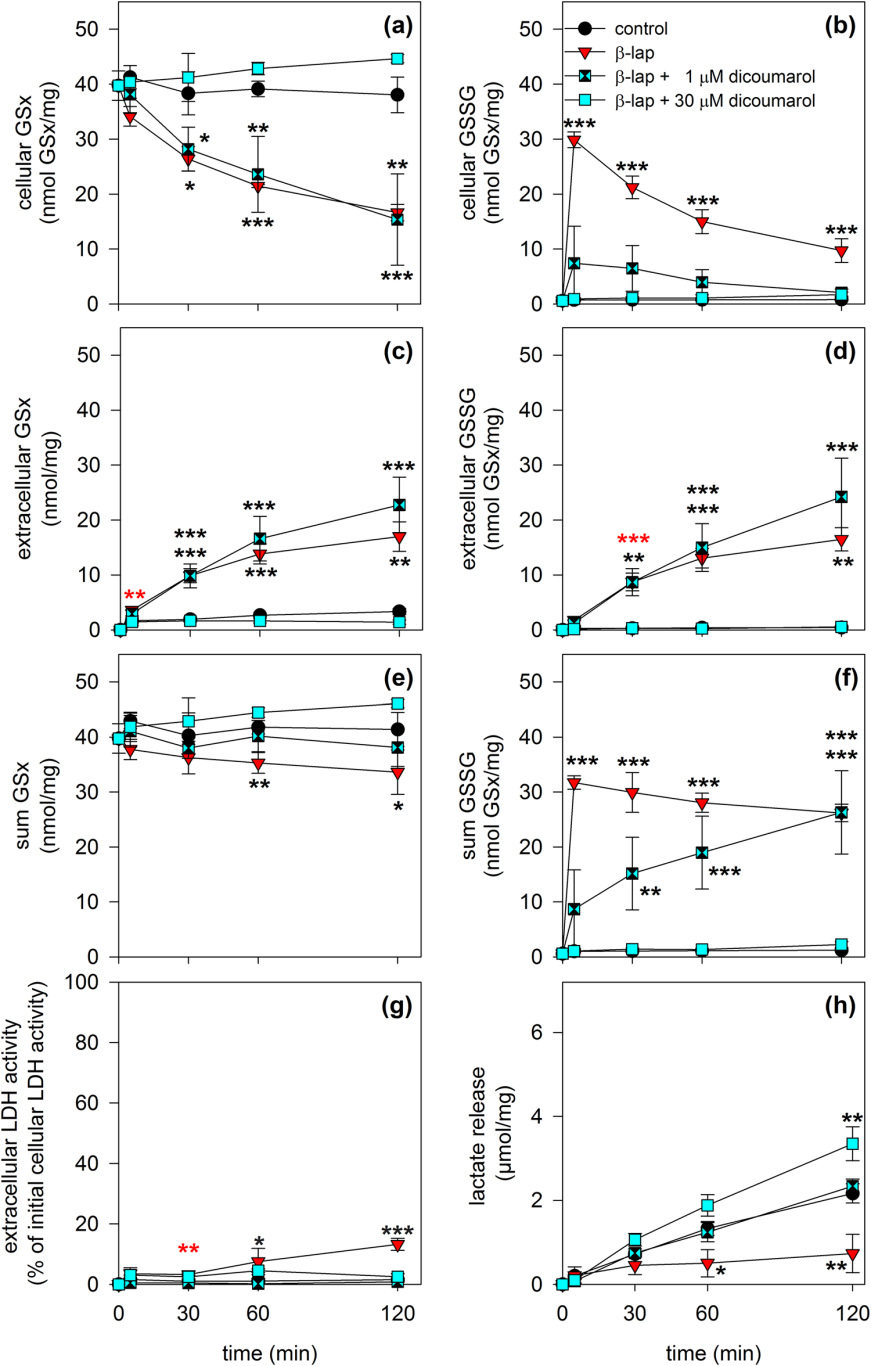
Impact of dicoumarol on the β-lap-induced GSH oxidation in cultured astrocytes. The cells were incubated without (control), with 20 µM β-lap alone, with 20 µM β-lap plus 1 µM dicoumarol or with 20 µM β-lap plus 30 µM dicoumarol for up to 120 min. For the indicated time points the intracellular GSx (**a**) and GSSG (**b**) contents, the extracellular GSx (**c**) and GSSG (**d**) contents as well as the sum of intra- plus extracellular GSx (**e**) and GSSG (**f**) contents of the cultures were determined, as well as the extracellular LDH activity (**g**) and the extracellular lactate concentration (**h**). The data shown are means ± SD of values obtained in 3 experiments on independently prepared cultures (n = 3). The initial specific GSx content of the cultures was 40 ± 3 nmol/mg and the initial specific GSSG content was 1 ± 0 nmol/mg. The protein content of the cultures was 137 ± 5 µg/well. The significance of differences (ANOVA) of data compared to the data obtained for control cells is indicated by asterisks (*p < 0.05, **p < 0.01, ***p < 0.001)

Incubation of astrocytes with 20 µM β-lap in the absence of dicoumarol affected the cellular and extracellular GSx and GSSG contents as well as the cells viability (Fig. 5) as already described for this condition above (Figs. 2, 4). If 1 µM dicoumarol was present during the incubation of astrocytes with β-lap, the rapid accumulation of cellular GSSG in β-lap-treated cells (88% of GSx after 5 min) was found strongly reduced (19% of GSx after 5 min; Fig. 5b), while the extracellular accumulation of GSSG was not altered compared to the incubation with β-lap alone (Fig. 5d). The sum of cellular plus extracellular GSx was hardly affected by the additional presence of 1 µM dicoumarol (Fig. 5e), while the sum of cellular plus extracellular GSSG values increased slowly over the incubation period of 2 h to values also found for astrocytes that had been exposed to β-lap alone (Fig. 5f). In addition, the presence of dicoumarol in a concentration of 1 µM prevented the β-lap-induced loss in cell viability (Fig. 5g) and the impaired lactate production (Fig. 5h).

If 30 µM dicoumarol was present during the incubation of astrocytes with β-lap, the cellular GSx content was slightly increased in comparison to the control incubation (Fig. 5a), while the extracellular accumulation of GSx (Fig. 5c), GSSG (Fig. 5d) and the sum of cellular plus extracellular GSx (Fig. 5e) was completely prevented. Importantly, presence of 30 µM dicoumarol also prevented the β-lap-induced transient cellular accumulation of GSSG (Fig. 5b) and LDH release (Fig. 5g) and restored the glycolytic lactate production which was found to be impaired in cells that had been treated with β-lap alone (Fig. 5h). In conclusion, the β-lap-induced impairments of cell viability and glycolytic metabolism was prevented by the presence of either 1 or 30 µM dicoumarol (Fig. 5), while 30 µM of dicoumarol had to be present to abolish the cellular and extracellular accumulation of GSSG in β-lap-induced astrocytes.

### β-Lap Induces ROS Formation in Astrocytes

**Fig. 6:**
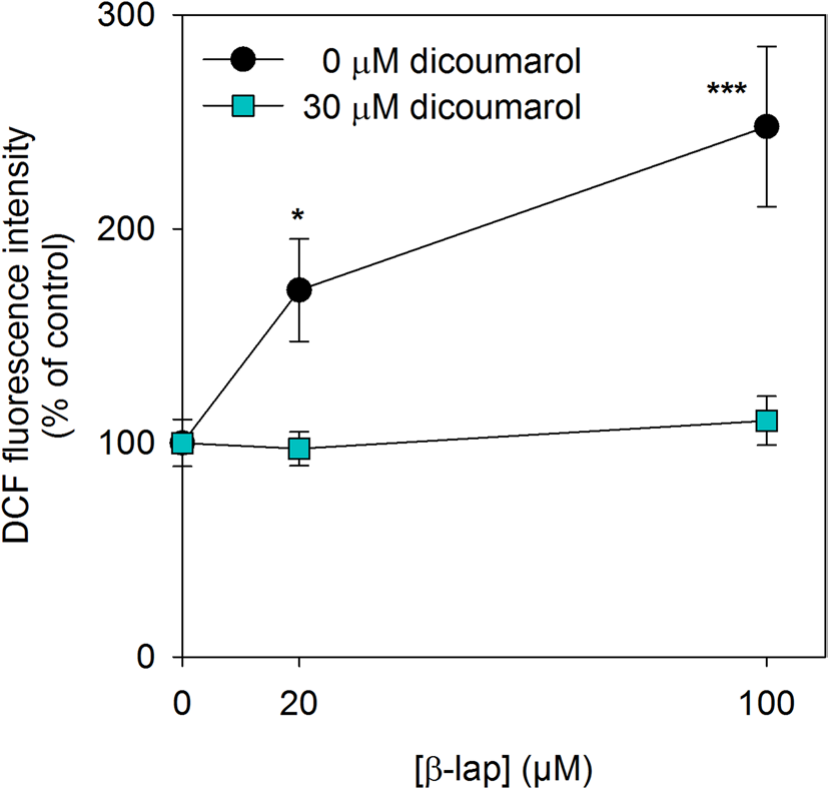
Test for ROS production in β-lap-treated astrocytes. Cultured astrocytes were loaded for 30 min with 50 µM DCFH_2_-DA and then incubated for 5 min without or with 20 or 100 µM β-lap in the absence or the presence of 30 µM dicoumarol. Subsequently, the cells were lysed and the DCF fluorescence was quantified in the lysate supernatant. The data shown are means ± SD of relative values (normalised to the control: incubation in the absence of β-lap) obtained in 3 experiments performed on independently prepared cultures (n = 3). Significant differences (ANOVA) between data obtained for incubations in the absence (0 µM) and the presence of β-lap are indicated by asterisks (*p < 0.05, ***p < 0.001)

To demonstrate ROS formation in β-lap-treated astrocytes, the cultures were loaded with DCFH_2_-DA before β-lap was applied. Quantification of cellular DCF fluorescence revealed that astrocytes that had been exposed for 5 min to β-lap in concentrations of 20 µM or 100 µM contained DCF contents that were increased by 70% and 150%, respectively (Fig. 6) compared to control cells (absence of β-lap). These increases in DCF fluorescence in β-lap-treated astrocytes were completely prevented, if astrocytes had been incubated with β-lap in the presence of 30 µM dicoumarol (Fig. 6).

### Reversibility of β-Lap Induced GSSG Accumulation

**Fig. 7:**
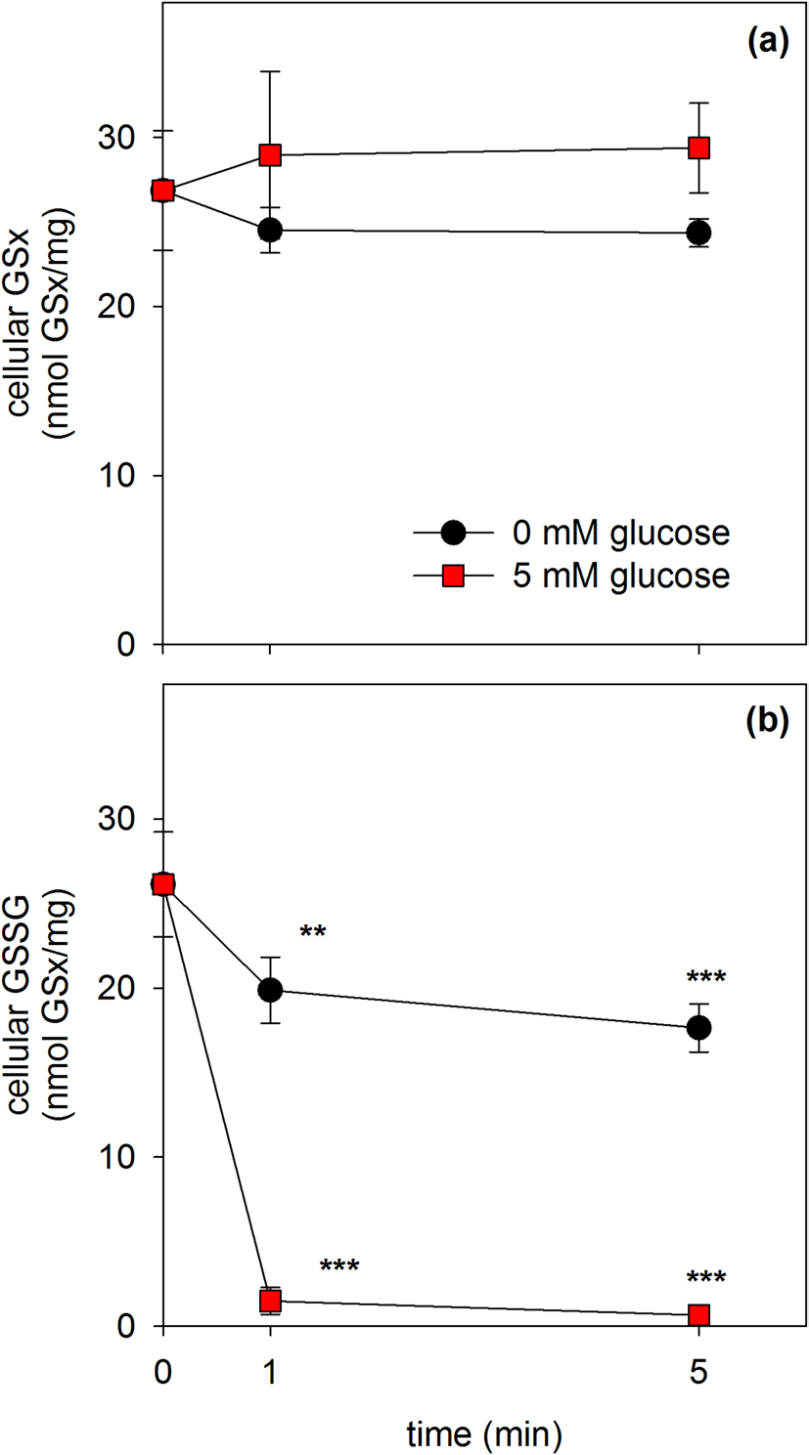
Reduction of the high β-lap-induced cellular GSSG levels after removal of β-lap. Astrocytes were preincubated for 20 min without glucose and subsequently for additional 10 min without glucose in the presence of 20 µM β-lap. After this preincubation, β-lap was removed by washing and the cells were incubated in the absence or the presence of 5 mM glucose for up to 5 min before cellular GSx (**a**) and GSSG (**b**) contents were determined. The initial specific GSx and GSSG contents of the cultures were 42 ± 1 nmol/mg and 1 ± 0 nmol/mg, respectively. The protein content of the cultures was 132 ± 6 µg/well. The data shown are means ± SD of values obtained in 3 experiments performed on independently prepared cultures (n = 3). Significant differences (ANOVA) compared to the data determined for the onset of the main incubation (t = 0 min) are indicated by asterisks (**p < 0.01, ***p < 0.001)

To test for the capacity of astrocytes to regenerate the normal high GSH to GSSG ratio after removal of β-lap and to investigate the importance of the availability of glucose for this process, the cells were deprived of glucose for 20 min and pre-incubated for 10 min with 20 µM β-lap in glucose-free IB to induce cellular GSSG accumulation. Subsequently, the β-lap was removed by washing and the cells were incubated for 1 min or 5 min without or with 5 mM glucose before the contents of cellular GSx and GSSG were determined. After the pre-incubation with β-lap the cellular GSx represented almost exclusively GSSG (Fig. 7) and a high GSSG to GSx ratio remained during a subsequent incubation in the absence of glucose (Fig. 7). In contrast, already after 1 min of incubation of β-lap-preincubated cells in the presence of glucose the cellular GSx contents (Fig. 7a) represented almost exclusively GSH as GSSG accounted to only 5% (1 min) and 2% (5 min) of the respective cellular GSx content (Fig. 7b).

### Consequences of an Application of Dicoumarol and/or SOD Plus Catalase on the GSSG Content in β-Lap-Treated Astrocytes

**Fig. 8:**
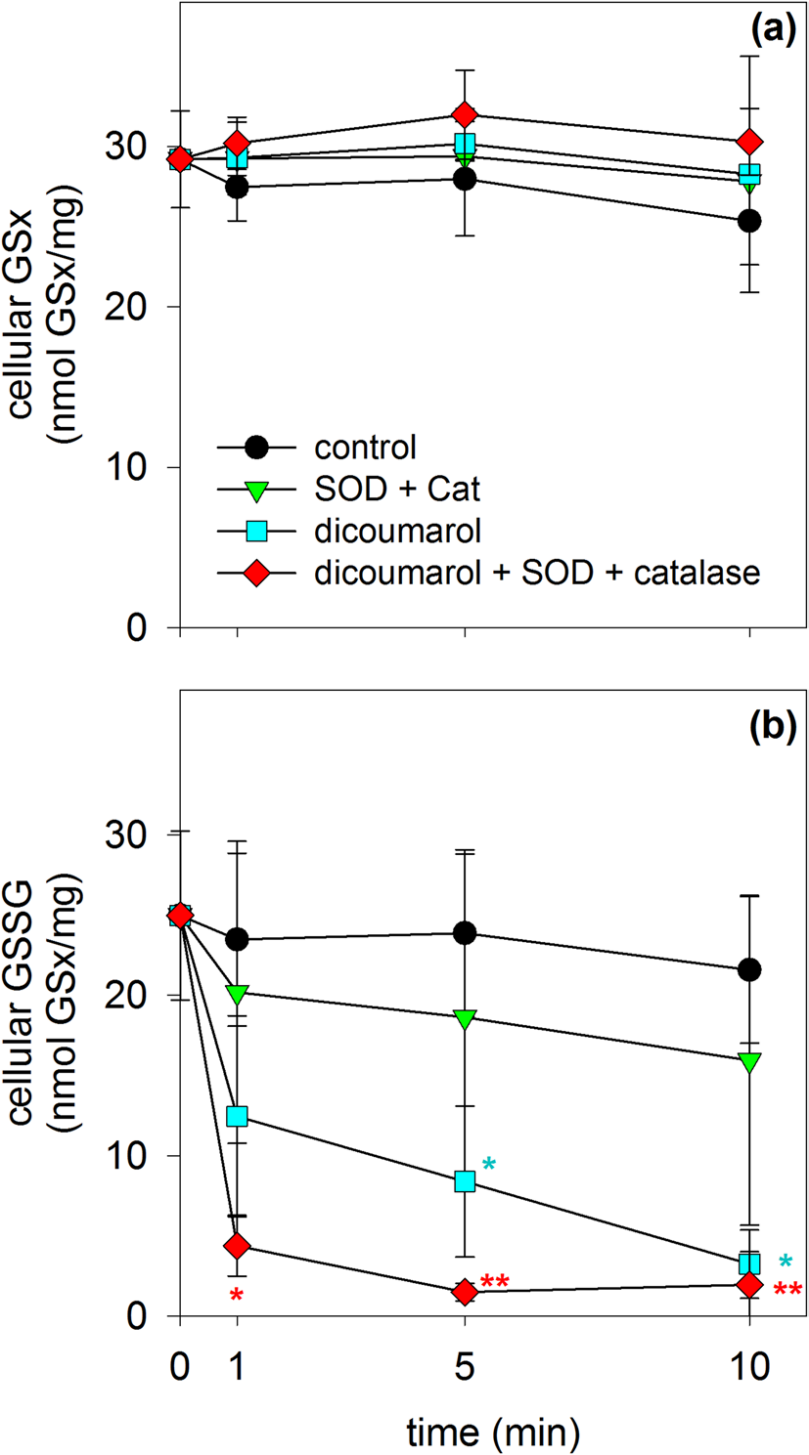
Consequences of an application of dicoumarol, SOD and catalase on the GSx and GSSG contents of β-lap-treated cultured astrocytes. Astrocytes were preincubated for 10 min with 20 µM β-lap before dicoumarol (final concentration of 30 µM) and/or SOD (100 U) plus catalase (260 U) were applied to the medium to start the main incubation of up to 10 min. For the indicated incubation periods the cellular GSx (**a**) and GSSG (**b**) contents were determined. The data shown are means ± SD of values obtained in 3 experiments performed on independently prepared cultures (n = 3). The initial specific GSx content of the cultures was 40 ± 2 nmol/mg, the initial specific GSSG content was below the detection limit of the assay used. The protein content of the cultures was 127 ± 14 µg/well. Significant differences (ANOVA) compared to the data obtained for the control condition (application of solvent) are indicated by asterisks written in the colours of the respective symbols (*p < 0.05, **p < 0.01)

To test whether application of dicoumarol is able to lower the high GSSG to GSx ratio found for β-lap-pretreated astrocytes, the cells were pre-incubated for 10 min with 20 µM β-lap which caused a high level of cellular GSSG (Fig. 8b). Application of a small volume of the solvent (IB), did not affect the high GSSG (Fig. 8b) to GSx (Fig. 8a, control) ratio during a subsequent incubation of up to 10 min. In contrast, application of dicoumarol to a final concentration of 30 µM lowered the high initial cellular GSSG content already by around 60% within 1 min and after 10 min of incubation hardly any GSSG was detectable in the cells (Fig. 8b). In contrast, application of superoxide dismutase (SOD, EC 1.15.1.1) plus catalase (EC 1.11.1.6) had hardly any effect in lowering the cellular GSSG content of β-lap-exposed cells. However, application of dicoumarol plus the enzymes to astrocytes that were exposed to β-lap almost completely restored the high GSH to GSSG ratio of the treated astrocytes within 1 min of incubation to levels that are similar to those of untreated cells (Fig. 8b). This suggests that the applied enzymes remove extracellular ROS which are present in β-lap-treated astrocyte cultures and contribute to the maintenance of a high cellular GSSH to GSH ratio.

### Test for the Suitability of β-Lap as Electron Cycler to Mediate Extracellular WST1 Reduction

**Fig. 9:**
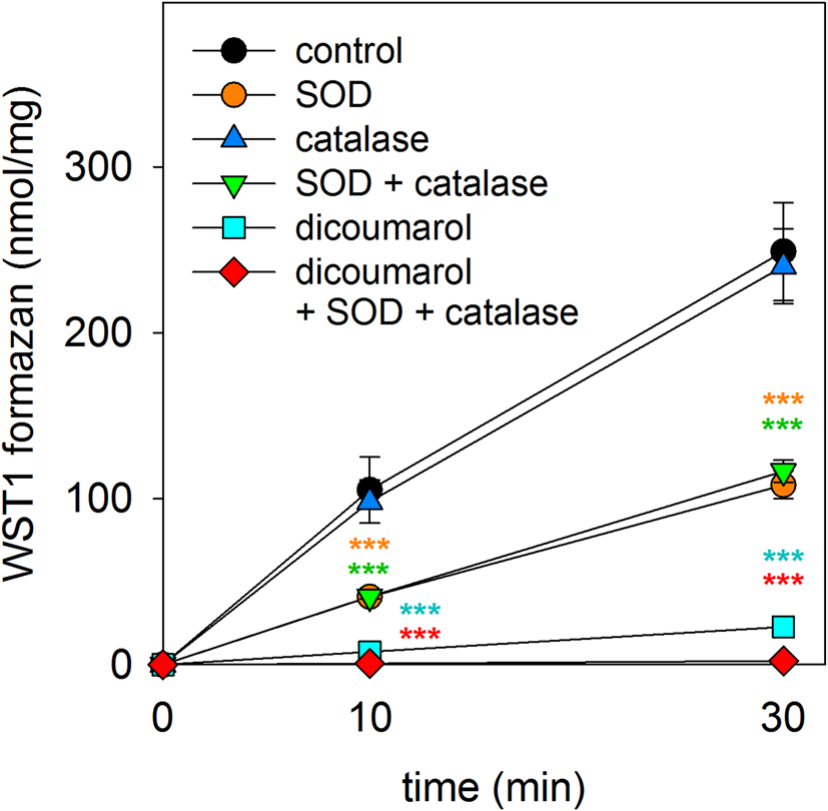
Use of β-lap as electron cycler to facilitate extracellular WST1 reduction by cultured astrocytes. The cells were incubated with 400 µM WST1 and 20 µM β-lap in the absence (control) or the presence of 30 µM dicoumarol, SOD (100 U) and/or catalase (260 U) for up to 30 min before the extracellular content of WST1 formazan was determined. The data shown are means ± SD of values obtained in 3 experiments performed on independently prepared cultures (n = 3). The protein content of the cultures was 148 ± 23 µg/well. Significant differences (ANOVA) compared to the data obtained for the control condition are indicated by asterisks written in the colours of the respective symbols (***p < 0.001)

The NQO1 substrate menadione has been reported to serve for cultured astrocytes as membrane permeable electron cycler that enables the transfer of electrons from intracellular sources for extracellular reduction of the membrane-impermeable tetrazolium dye WST1 [46]. Therefore, also β-lap was considered as potential electron cycler that can shuttle electrons in its labile reduced form from intracellular sources to extracellular WST1. To test for this option, astrocytes were incubated with 20 µM β-lap and 400 µM WST1 in glucose-containing IB for up to 30 min before the extracellular concentration of WST1 formazan was photometrically determined. Indeed, the presence of β-lap allowed efficient WST1 reduction as demonstrated by the strong increase in extracellular WST1 formazan content (Fig. 9).

To investigate which β-lapachol-derived ROS may be involved in extracellular WST1 reduction, the detoxifying enzymes catalase and/or SOD were applied. The β-lap-dependent WST1 reduction was almost completely prevented in the presence of dicoumarol alone or of dicoumarol plus catalase plus SOD. In the absence of dicoumarol the presence of SOD lowered extracellular WST1 reduction by around 50%, while catalase alone did not affect WST1 reduction and catalase in combination with SOD did not enhance the observed effect of SOD alone (Fig. 9).

## Discussion

Exposure of cancer cells to β-lap has been reported to induce toxicity and apoptosis [3–8, 13] which involves intracellular superoxide formation and oxidative stress [47] due to futile redox cycling by NQO1 [10, 13, 47]. As also astrocytes contain NQO1 [35] the consequences of an exposure of astrocyte cultures to β-lap was investigated. Presence of β-lap in concentrations above 10 µM caused within minutes a rapid concentration-dependent ROS formation and GSH oxidation that was followed by a slower impairment of glycolytic lactate production and finally in compromised cell membrane integrity. NQO1 is able to use both NADH and NADPH as electron donor [50]. Thus, the slowed lactate production by astrocytes after application of higher concentrations of β-lap may be the consequence of a NQO1-dependent consumption of glycolytically generated NADH and of an accelerated metabolism of glucose-6-phosphate by the pentose phosphate pathway (PPP) to compensate for the NQO1-dependent consumption of NADPH. Furthermore, lowered NAD^+^ levels due to the activation of poly-(ADP-ribose) polymerase (PARP) during acute oxidative stress as shown previously [13], may also contribute to the lower glycolytic activity in β-lap-treated astrocytes.

All β-lap-induced adverse effects on cultured astrocytes were completely prevented by the NQO1 inhibitor dicoumarol [48, 49], consistent with the view that NQO1 plays the central role in generating β-lap-induced oxidative stress [13, 47]. Concerning the potential application of β-lap as anti-tumor treatment [7, 14], it should be considered that also normal tissue cells which contain substantial NQO1 activity, such as brain astrocytes, may be affected by β-lap-induced oxidative stress.

Recently we have reported that the quinone and NQO1 substrate menadione induces oxidative stress and rapid GSH oxidation in cultured astrocytes [25]. However, the consequences of a menadione exposure of astrocytes cannot be prevented by application of the NQO1 inhibitor dicoumarol due to NQO1-independent ROS formation by menadione [25]. This strongly contrast to the results observed for β-lap-treated astrocytes, where ROS formation and GSSG accumulation were completely prevented by dicoumarol, suggesting that the oxidative stress observed in β-lap-treated astrocytes exclusively depends on the activity of NQO1. Thus, the sequential application of β-lap and dicoumarol displays a suitable experimental system to rapidly induce (application of β-lap) and terminate (addition of dicoumarol) the duration of an acute oxidative stress condition, at least for cultured astrocytes. Such an experimental paradigm that clearly defines an experimental setting for studying consequences of oxidative stress might also by suitable for other types of cultured cells that contain substantial activities of NQO1. However, it should be considered that such reactions lead to an excessive consumption of reduced nicotinamide coenzymes, NADH and NADPH, which may affect metabolic and protective pathways during the treatment.

For cell lysates of astrocytes dicoumarol has been shown to inhibit NQO1 activity with half-maximal inhibition in the nM range [39]. However, for intact astrocytes even at a concentration of 1 µM extracellular dicoumarol was unable to completely inhibit NQO1-dependent β-lap-mediated GSH oxidation, as evident by the small but significant increase in the GSSG to GSH ratio of astrocytes that had been exposed to β-lap plus dicoumarol for 5 min. Most likely higher extracellular concentrations of dicoumarol have to be applied to generate a sufficiently high intracellular concentration of dicoumarol to completely inhibit NQO1. This was achieved by the application of 30 µM dicoumarol which completely prevented NQO1-dependent β-lap-mediated ROS formation and GSSG accumulation. However, application of such high micromolar concentrations of dicoumarol has the disadvantage that also the export of GSH, GSSG and GSH-conjugates from astrocytes via Mrp1 is inhibited [26].

**Fig. 10:**
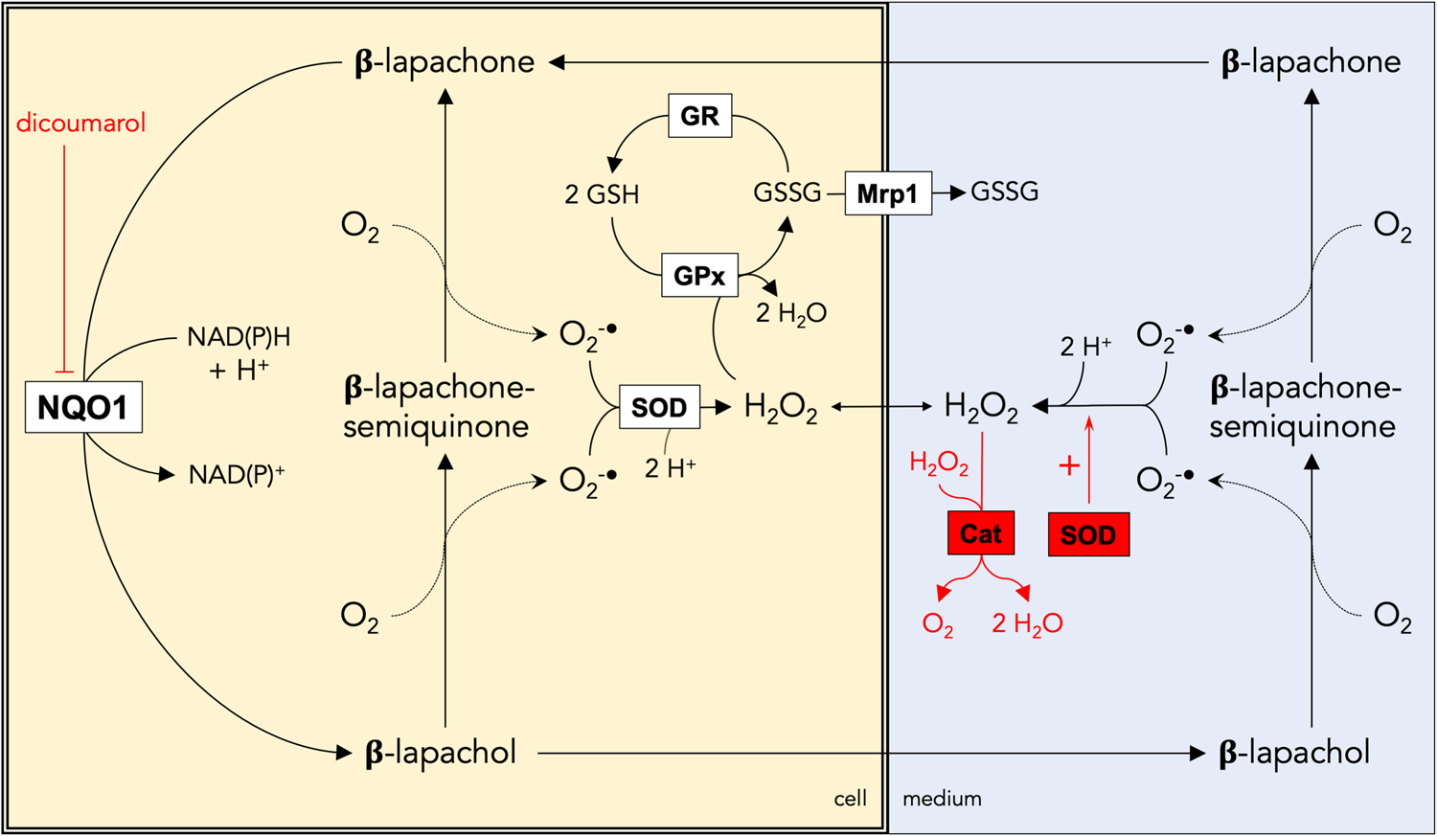
Consequences of a treatment of astrocytes with β-lap. β-lap is reduced within astrocytes by the dicoumarol-sensitive NQO1 in a two-electron transfer reaction to β-lapachol. The labile β-lapachol can auto-oxidise inside of the cell in two distinct oxidation steps first to β-lapachone-semiquinone and then to β-lapachone, thereby producing 2 molecules of superoxide. Superoxide is rapidly disproportionated by superoxide dismutase (SOD) to oxygen and H_2_O_2_. Cellular reduction of H_2_O_2_ by glutathione peroxidase (GPx) leads to the oxidation of GSH and the formation of cellular GSSG which can subsequently be reduced to GSH by the NADPH-dependent glutathione reductase (GR) or be released from the cells via multidrug resistance protein 1 (Mrp1). β-lapachol is membrane-permeable and can be released from astrocytes. Auto oxidation of extracellular β-lapachol will generate extracellular superoxide which can either mediate WST1 reduction or can chemically disproportionate to H_2_O_2_. This H_2_O_2_ can enter the astrocyte and can be detoxified by the astrocytic GPx. Application of the NQO1 inhibitor dicoumarol, prevents all effects observed for a treatment of astrocytes with β-lap, demonstrating the central function of NQO1 in generating the β-lap-induced oxidative stress. Application of SOD and catalase (Cat) to β-lap-treated cells rapidly removes extracellular superoxide and H_2_O_2_, thereby preventing cellular GSH oxidation caused by GPx-mediated reduction of extracellular H_2_O_2_ that was taken up by the cells

The cellular consequences of an application of β-lap to cultured astrocytes that lead to ROS formation and GSSG oxidation are schematically shown in Fig. 10. Reduction of β-lap by NQO1 generates the instable β-lapachol which generates two molecules of superoxide during its auto-oxidation to β-lap [13, 51]. In cells, superoxide is rapidly disproportionated by SODs to oxygen and H_2_O_2_ [52] and the peroxide can be reduced to water by GPx which takes the electrons required from GSH and generates GSSG [17]. Cellular GSSG is reduced to GSH in the reaction catalysed by GR which uses NADPH as electron source [53]. For β-lap-treated astrocytes a strong accumulation of GSSG was observed, indicating that for those conditions the rate of GSH oxidation by GPx is strongly exceeding the rate of GR-mediated GSSG reduction as previously reported for astrocytes that had been exposed to acute or chronic H_2_O_2_-stress [23, 24]. One consequence of the strong accumulation of GSSG in β-lap-treated astrocytes is the export of GSSG, which is mediated by Mrp1 [30, 33] and has previously been reported for conditions that induce severe oxidative stress in cultured astrocytes [25, 26, 30, 33].

Upon termination of the β-lap-induced oxidative stress, either by removal of β-lap or by inhibiting NQO1-mediated β-lap reduction by application of dicoumarol, the level of cellular GSSG declined rapidly and the normal high ratio of GSH to GSSG was re-established within minutes, demonstrating the high capacity of astrocytes to efficiently reduce GSSG by the GR reaction. The rapid GSSG reduction was strongly impaired in glucose-deprived astrocytes, confirming the reported importance of the glucose metabolism via the PPP for providing the NADPH required for GR-dependent GSSG reduction [17, 54]. The rapid regeneration of the high GSH to GSSG ratio in glucose-fed astrocytes demonstrates also that an irreversible inhibition of GR or of the NADPH-regeneration by the PPP which also would lead to a strong GSSG accumulation in β-lap-treated astrocytes can be excluded.

Although a rapid regeneration of cellular GSSG was observed after application of dicoumarol to the β-lap-containing incubation medium, this dicoumarol-initiated cellular GSSG reduction was accelerated by co-application of dicoumarol with SOD plus catalase. As these enzymes cannot penetrate an intact cell membrane, it was concluded that release of ROS and/or extracellular generation of ROS contribute to the oxidative stress generated after application of β-lap (Fig. 10). As shown for menadione [46], also the β-lap/β-lapachol redox pair was found to efficiently act as electron cycler and to mediate the electron transfer from cellular sources for extracellular WST1 reduction which requires export of β-lapachol and reuptake of β-lap (Fig. 10). As H_2_O_2_ does not chemically reduce WST1 (data not shown) and as the presence of SOD, but not of catalase, lowered the extracellular WST1-reduction in presence of β-lap by at least 50%, it was concluded that β-lapachol had been indeed released from β-lap-treated astrocytes and that this labile compound generated extracellularly superoxide which was used for WST1 reduction. However, as superoxide can be released from cells [55], we can currently also not exclude that intracellular β-lapachol-derived superoxide may be released from astrocytes and contribute to the observed extracellular WST1 reduction. In addition, extracellular H_2_O_2_ originating from cellular or extracellular β-lapachol-derived superoxide could have contributed to the slow regeneration of the normal high cellular GSH to GSSG ratio after uptake into the cells and subsequent cellular clearance via GSH and GPx after termination of the oxidative stress by application of dicoumarol. Thus, for efficient and immediate termination of β-lap-induced oxidative stress it is recommended to apply both dicoumarol and SOD plus catalase in order to inhibit the formation of new ROS and to eliminate the extracellular reservoir of ROS, respectively.

Unexpectedly, the extracellular lactate accumulation in the presence of β-lap and 30 µM dicoumarol was found elevated compared to the values obtained for control cells. Previous work from our group suggests that in cultured astrocytes NADPH may be the preferred electron donor to deliver electrons for NQO1-dependent reactions [39]. Assuming that the consumption of NADPH is lowered by dicoumarol-mediated inhibition of cytosolic NQO1, less glucose-6-phosphate would be needed as substrate for NADPH regeneration by the PPP [56] and more glucose-6-phosphate could be used for glycolytic lactate production. However, we can also not exclude a potential direct action of dicoumarol on mitochondrial processes which may lower mitochondrial ATP production and as a consequence increase glycolytic flux as previously shown for several compounds [57, 58].

In conclusion, application of micromolar concentrations of β-lap induces severe oxidative stress in cultured astrocytes, as evident by accelerated ROS production and strong GSSG accumulation. Such severe consequences are important to be considered for potential systemic application of β-lap as anti-tumor drug. Although data on the permeability of β-lap through the blood–brain barrier are missing so far [59], this compound is due to its hydrophobicity [2] quite likely to cross this barrier after systemic application and thereby is likely to interact with astrocytes in brain. As the β-lap-induced oxidative stress in cultured astrocytes depends exclusively on the activity of the enzyme NQO1 which can be efficiently inactivated by dicoumarol, the sequential application of β-lap and dicoumarol is a valuable experimental setup to rapidly induce and terminate an acute oxidative stress condition in NQO1 expressing cultured cells. This experimental setting could be useful for studying export of GSSG during oxidative stress, the cellular mechanisms required to regenerate GSH or the metabolic processes that provide the NADPH required for GSSG reduction after terminating the oxidative stress. With some adaptations, the experimental setting should also be applicable for studies of the consequences of oxidative stress in other types of cultured cells as long as these cells display sufficient activity of NQO1.
